# Associations Between Family Member Involvement and Outcomes of Patients Admitted to the Intensive Care Unit: Retrospective Cohort Study

**DOI:** 10.2196/33921

**Published:** 2022-06-15

**Authors:** Tamryn F Gray, Anne Kwok, Khuyen M Do, Sandra Zeng, Edward T Moseley, Yasser M Dbeis, Renato Umeton, James A Tulsky, Areej El-Jawahri, Charlotta Lindvall

**Affiliations:** 1 Department of Medicine Harvard Medical School Boston, MA United States; 2 Division of Palliative Medicine Brigham and Women's Hospital Boston, MA United States; 3 Department of Psychosocial Oncology and Palliative Care Dana-Farber Cancer Institute Boston, MA United States; 4 Department of Informatics & Analytics Dana-Farber Cancer Institute Boston, MA United States; 5 Department of Biological Engineering and Department of Mechanical Engineering Massachusetts Institute of Technology Cambridge, MA United States; 6 Department of Biostatistics Harvard T.H. Chan School of Public Health Boston, MA United States; 7 Department of Pathology and Laboratory Medicine Weill Cornell Medicine New York, NY United States; 8 Department of Medicine Massachusetts General Hospital Cancer Center Boston, MA United States

**Keywords:** critical care, natural language processing, family, electronic health records, goals of care, intensive care unit, ICU

## Abstract

**Background:**

Little is known about family member involvement, by relationship status, for patients treated in the intensive care unit (ICU).

**Objective:**

Using documentation of family interactions in clinical notes, we examined associations between child and spousal involvement and ICU patient outcomes, including goals of care conversations (GOCCs), limitations in life-sustaining therapy (LLST), and 3-month mortality.

**Methods:**

Using a retrospective cohort design, the study included a total of 858 adult patients treated between 2008 and 2012 in the medical ICU at a tertiary care center in northeastern United States. Clinical notes generated within the first 48 hours of admission to the ICU were used with standard machine learning methods to predict patient outcomes. We used natural language processing methods to identify family-related documentation and abstracted sociodemographic and clinical characteristics of the patients from the medical record.

**Results:**

Most of the 858 patients were White (n=650, 75.8%); 437 (50.9%) were male, 479 (55.8%) were married, and the median age was 68.4 (IQR 56.5-79.4) years. Most patients had documented GOCC (n=651, 75.9%). In adjusted regression analyses, child involvement (odds ratio [OR] 0.81; 95% CI 0.49-1.34; *P*=.41) and child plus spouse involvement (OR 1.28; 95% CI 0.8-2.03; *P*=.3) were not associated with GOCCs compared to spouse involvement. Child involvement was not associated with LLST when compared to spouse involvement (OR 1.49; 95% CI 0.89-2.52; *P*=.13). However, child plus spouse involvement was associated with LLST (OR 1.6; 95% CI 1.02-2.52; *P*=.04). Compared to spouse involvement, there were no significant differences in the 3-month mortality by family member type, including child plus spouse involvement (OR 1.38; 95% CI 0.91-2.09; *P*=.13) and child involvement (OR 1.47; 95% CI 0.9-2.41; *P*=.12).

**Conclusions:**

Our findings demonstrate that statistical models derived from text analysis in the first 48 hours of ICU admission can predict patient outcomes. Early child plus spouse involvement was associated with LLST, suggesting that decisions about LLST were more likely to occur when the child and spouse were both involved compared to the involvement of only the spouse. More research is needed to further understand the involvement of different family members in ICU care and its association with patient outcomes.

## Introduction

### Background

Mechanically ventilated critically ill patients often lack decisional capacity [1-3] and rely on family members for their care and medical decision-making [2-6]. In the critical care environment, where decisions about tests, procedures, and treatments must be made quickly [7,8], physicians turn to surrogate decision makers for guidance about goals of care and making decisions to limit life-sustaining treatment [1,6,7,9-11]. Critical care organizations have strongly encouraged a family-centered approach to care [12,13]; however, information about when, how, and which family members are engaged over the course of illness remains poorly understood [7].

Although clinicians often expect 1 family member to be the “voice” for the patient, several family members are often involved [14,15]. In the event that the patients no longer possess the requisite capacity to make their own health care decisions or are too ill, which is common in the intensive care unit (ICU) setting [16], the health care proxy is the most common way through which patients appoint a surrogate decision-maker to make decisions on their behalf [17]. Typically, the health care provider has a priority list of individuals to be designated for this role, and at the top of the hierarchy is often the patient’s spouse followed by the adult child/children, parents, and adult sibling(s) [18,19]. In American families, the spouse is commonly the first in line to assume the role of a health care proxy [20] and is informed if he or she is aware of (1) the patient’s personal definition of quality of life, (2) his or her specific plan if he or she cannot achieve this quality of life, and (3) desired location of death [21]. If no spouse is available to provide care, adult children often take on the role and sometimes share care tasks [22]. Although studies examining family members in the ICU have focused on family needs, communication, and satisfaction with care [23-27], to our knowledge, no studies have discerned the distinct involvement of spouses and children in care decisions and its impact on patient outcomes in the medical ICU (MICU) setting.

### Objective

We sought to describe family member involvement in decision-making, by relationship status, for patients treated in the ICU. We also examined patient characteristics associated with child and spousal involvement. Using documentation of family interactions in clinical notes, we examined the association between child and spousal involvement in the first 48 hours of admission and ICU patient outcomes, including goals of care conversations (GOCCs), limitations in life-sustaining therapy (LLST), and mortality.

## Methods

### Data Source

Our data source was the Medical Information Mart for Intensive Care III (MIMIC-III) database, developed by the Massachusetts Institute of Technology (MIT) and Beth Israel Deaconess Medical Center (BIDMC), and it is a large, freely available database. The MIMIC database provided deidentified demographic, administrative, clinical, and survival outcome data for all adult ICU admissions at the BIDMC [28]. For our analysis, we used data between 2008 and 2012 to include clinical notes from a broad group of clinicians likely to document engagement with patients’ families, including physicians, nursing staff, social workers, case managers, and physician assistants [29]. The Institutional Review Board of the BIDMC and MIT approved use of the MIMIC-III database by any investigator who fulfills data-user requirements [29]. This research was deemed exempt by the Dana-Farber Cancer Institute Institutional Review Board (approval number 18-192).

### Study Population

**Figure 1:**
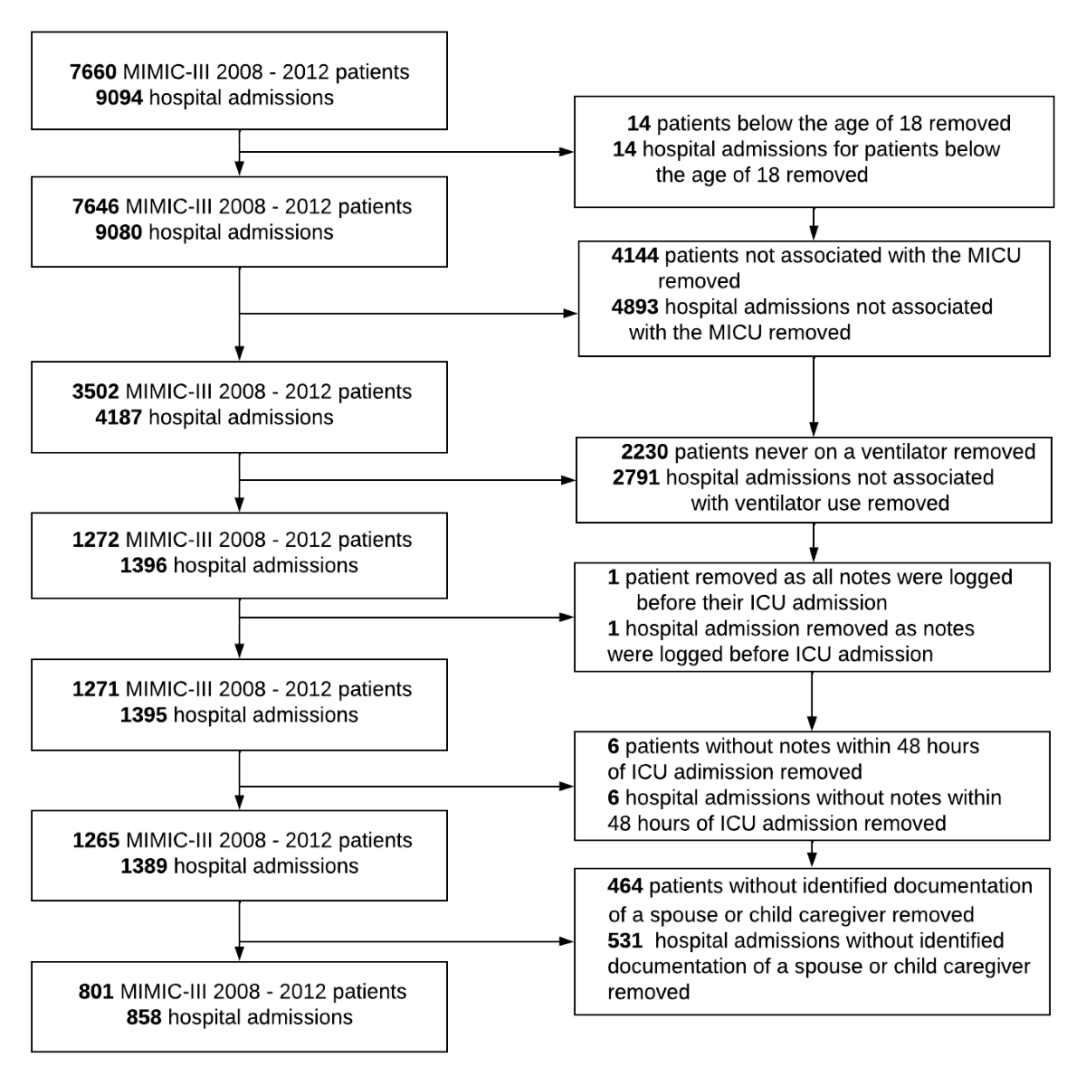
Flow diagram showing patient selection in the study. ICU: intensive care unit; MIMIC-III: Medical Information Mart for Intensive Care III. It is a deidentified demographic, administrative, clinical, and survival outcome database for adult ICU admissions. MICU: medical intensive care unit.

The study population included patients at least 18 years of age who were treated in the MICU at the BIDMC in Boston between 2008 and 2012 (Figure 1). We focused exclusively on MICU patients commonly facing life-threatening conditions that may warrant family involvement in decision-making [30]. We excluded patients with an ICU length of stay (LOS) less than 48 hours and those lacking available clinical notes due to potential privacy disclosures (eg, VIPs). For patients with multiple ICU admissions during a single hospitalization, only the first admission was used for analysis.

### Natural Language Processing (NLP)

Family communication is often recorded as free text in the clinical notes [31]. Manual abstraction of these data is time-consuming and prone to human error, thus benefiting from a structured approach using standard NLP methods [31]. The ability of NLP methods to identify electronic health record (EHR) documentation of family involvement in the ICU was evaluated using a multistep process. First, we constructed a keyword library to develop a standard structure, including typographical errors that might be present. We used the text annotation software, ClinicalRegex [32], to identify documentation of child and spousal or partner involvement in the EHR (referred to as “family involvement”). ClinicalRegex was developed by the Lindvall Lab at Dana-Farber Cancer Institute and has been applied in multiple studies [32-35] to identify defined keywords or phrases within clinical notes, accounting for varieties in language, spelling, and punctuation. Using a predefined ontology, the software displayed clinical notes that contain the highlighted keywords or phrases associated with family. Our ontology contained two domains of documentation regarding family involvement: (1) spouse or partner and (2) children. The keyword library was refined to prioritize sensitivity over specificity and validated by expert review of a random selection of notes identified by the library as well as manual review of notes not identified by the library. The final keyword library is provided in Multimedia Appendix 1.

Second, once the ontology was developed, independent coders (TFG, KMD, and SZ) reviewed a subset of 100 random samples of charts in ClinicalRegex using the keyword library to examine whether each clinical note contained keywords related to family involvement. Human experts labeled notations using prespecified codes (eg, using “0” to label notations where keywords appeared out of context for exclusion or using “1” to label notations for inclusion), and the presence or absence of family-related documentation was determined at the hospital admission level. Interrater agreement was excellent (κ values of 0.83 and 0.82 for child and spouse, respectively).

### Study Measures

#### Family Involvement

To identify family-related documentation in the EHR, we first conducted a literature search of relevant keywords related to spouse and child [22,36,37]. For our keyword library, we developed an extensive list to account for the wide variation in describing spouse and child. For example, spouse was described as husband, wife, fiancé, girlfriend, boyfriend, companion, partner, spouse, comate, etc. Child was described as son, daughter, grandchild, teenage, girl, boy, child, children, grandson, granddaughter, etc. Multimedia Appendix 1 presents the exact phrases used in the keyword bank. Multimedia Appendices 2 and 3 respectively describe examples of how the keywords found in the clinical notes were used in the relevant context as well as the keywords that were not used in the analysis because they were used in a nonrelevant context.

#### Sociodemographic and Clinical Factors

We collected demographic information (admission age, sex, race, ethnicity, and marital status) as well as clinical characteristics including the sequential organ failure assessment score (SOFA) and Elixhauser Comorbidity Index. The SOFA score described the time course of multiple organ dysfunction using a limited number of routinely measured variables [38], and the Elixhauser Comorbidity Index quantified the effect of comorbidities on patient outcomes [39]. The sociodemographic and clinical characteristics of the patients were ascertained by EHR data extraction.

#### Health Care Usage

For health care usage outcomes, the discharge location was included (eg, home, home health care, hospice, short-term hospital, long-term-care hospital, skilled nursing facility [SNF], “other facilities,” and in-hospital death). The LOS for obtaining the hospitalization index and hospital readmission were also determined for each patient. For our analyses, home was defined as either home or home health care. Facility was defined as either hospice, short-term hospital, long-term care, SNF, or “other facilities.”

### Outcome Measures

#### GOCC Documentation

The National Quality Forum recommends that GOCCs be documented in the EHR within the first 48 hours of an ICU admission, especially among frail and seriously ill patients. For our study, we identified GOCCs using an operational definition previously described elsewhere [29]. GOCC documentation required both of the following details: (1) mention of a conversation with either the patient or a family member and (2) mention of a specific care preference pertaining to hospital care [29]. Ascertained by free-text data in the clinical notes, GOCC documentation included discussion about advance care planning activities (values, goals, and preferences considering future care), completion of advance directives or Physician Order for Life-Sustaining Treatment forms, or referral to hospice or subspecialty palliative care services [40].

#### LLST Conversations

Similar to our previous study [29] and other research [41], LLST included documentation from free-text data within clinical notes regarding a do-not-resuscitate or do-not-intubate (DNR/DNI) code status, LLST, acknowledgment of patient or family wishes to decline any interventional procedures (including central venous line, temp wire placement, etc) but agreement for medical management, preference for no heroic measures, no blood transfusions, no resuscitations, and no blood pressure interventions.

#### Mortality

To assess the 3-month mortality since hospital admission, we used a binary outcome of died and not died within 3 months since hospital admission based on EHR review.

### Statistical Analysis

We used descriptive statistics to summarize the sample, including the sociodemographic and clinical characteristics of the patients as well as health care use and mortality. We performed univariate analyses to assess the relationships between the sociodemographic and clinical characteristics of the patients and family involvement, stratified by the type of family member (overall cohort, both child and spousal involvement, child only involvement, and spouse only involvement). To assess the independent associations between family involvement and GOCC, LLST, and 3-month mortality, we developed multivariable logistic regression models. For each dependent variable, separate models were fitted, adjusting for sex, marital status, race and ethnicity, age, SOFA, and Elixhauser scores identified a priori based on prior literature [22,38,42,43]. All statistical tests and CIs, as appropriate, were performed as 2-sided tests, and all reported *P* values <.05 were considered statistically significant. We performed all statistical analyses using Python version 3.7.6 and library statsmodels version 0.12.0.

## Results

### Patient Characteristics

Table 1 describes the sociodemographic and clinical characteristics of the patients at hospital admission (N=858). The median age was 68 (IQR: 57-79) years, most patients were non-Hispanic White (n=650, 75.8%), and approximately half were male (n=437, 50.9%) and married (n=479, 55.8%). The median SOFA and Elixhauser scores were 6 (IQR 4-9) and 5 (range 3-6), respectively. The median LOS was 9 (IQR 4.9-16.8) days. More than a quarter of these patients died in the ICU (n=253, 29.5%), whereas the majority were either discharged to a facility or home (n=379, 44.2% and n=223, 26%, respectively). When compared to child plus spouse involvement and spouse only involvement, patients with child only involvement (n=352) were more likely to be female (235/352, 66.8%), not married or partnered (265/352, 75.3%), and older (median age of 76.7 [IQR 66-85] years) (Table 1). When both spouse and child were involved (n=202), patients were mostly male (123/202, 60.9%), married (170/202, 84.2%), and had a median age of 70 (range 61-77) years. In comparison with White patients, non-White patients had a high proportion of child only involvement (95/165, 57.6% vs. 242/650, 37.2%).

**Table 1:** Patient characteristics^a^.

Characteristics		Overall (N=858)	Both (n=202)	Child (n=352)	Spouse (n=304)	*P* value
**Sex, n (%)**						<.001
	Male	421 (49.1)	79 (39.1)	235 (66.8)	107 (35.2)	
	Female	437 (50.9)	123 (60.9)	117 (33.2)	197 (64.8)	
**Marital status, n (%)**						<.001
	Married	479 (55.8)	170 (84.2)	72 (20.5)	237 (78.0)	
	Not married	354 (41.3)	27 (13.4)	265 (75.3)	62 (20.4)	
	Unknown	25 (2.9)	5 (2.5)	15 (4.3)	5 (1.6)	
**Ethnicity, n (%)**						<.001
	White (non-Hispanic)	650 (75.8)	163 (80.7)	242 (68.8)	245 (80.6)	
	Other	165 (19.2)	29 (14.4)	95 (27.0)	41 (13.5)	
	Unknown	43 (5.0)	10 (5.0)	15 (4.3)	18 (5.9)	
Admission age in years, median (IQR)		68.4 (56.5-79.4)	69.7 (61-77.4)	76.7 (66-85)	58.4 (48.4-67)	<.001
Hospital LOS^b^ in days, median (IQR)		9 (4.9-16.8)	8.6 (4.7-16.1)	8 (4.7-14.7)	12.1 (6-21.1)	<.001
**Discharge status, n (%)**						<.001
	Death	253 (29.5)	81 (40.1)	109 (31.0)	63 (20.7)	
	Facility	379 (44.2)	85 (42.1)	158 (44.9)	136 (44.7)	
	Home	223 (26.0)	36 (17.8)	84 (23.9)	103 (33.9)	
	Unknown	3 (0.3)	0 (0)	1 (0.3)	2 (0.7)	
**Mortality, n (%)**						<.001
	In-hospital mortality	253 (29.5)	81 (40.1)	109 (31.0)	63 (20.7)	
	3 months from hospital admission	342 (39.9)	98 (48.5)	152 (43.2)	92 (30.3)	
	1 year from hospital admission	442 (51.5)	118 (58.4)	198 (56.2)	126 (41.4)	
	6 months from ICU^c^ discharge	397 (46.3)	108 (53.5)	173 (49.1)	116 (38.2)	
Readmission, n (%)		196 (22.8)	43 (21.3)	90 (25.6)	63 (20.7)	.28
Documented goals of care conversation, n (%)		651 (75.9)	164 (81.2)	266 (75.6)	221 (72.7)	.09
Documented conversations about limitations in code status, n (%)		274 (31.9)	73 (36.1)	149 (42.3)	52 (17.1)	<.001
SOFA^d^ score, median (IQR)		6 (4-9)	7 (5-10)	6 (4-9)	5.5 (3-8)	<.001
Elixhauser score, median (IQR)		5 (3-6)	5 (3-6)	5 (3-6)	4 (3-6)	.06

^a^Patient characteristics of study the cohort were stratified by documentation of family involvement. For discharge status, chi-square tests may not be valid due to a low number of examples in some categories.

^b^LOS: length of stay.

^c^ICU: intensive care unit.

^d^SOFA: sequential organ failure assessment score.

### Association Between Family Involvement and GOCC

Overall, most patients had documented GOCC (651/858, 75.9%) (Table 1). Child involvement (odds ratio [OR] 0.81; 95% CI 0.49-1.34; *P*=.41) and involvement of child plus spouse (OR 1.28; 95% CI 0.8-2.03; *P*=.3) were not associated with GOCC when compared to spouse only involvement (Table 2).

**Table 2:** Goals of care conversations^a^.

Variables		Odds ratio (95% CI)		*P* value
**Sex (reference group: male)**				
	Female	1.18 (8.84-1.65)	.35	
**Marital status (reference group: married)**				
	Not married	1.19 (0.79-1.78)	.41	
	Unknown	1.09 (0.4-2.96)	.86	
**Ethnicity (reference group: White)**				
	Other	0.8 (0.54-1.2)	.28	
	Unknown	0.99 (0.46-2.13)	.97	
**Type of family member documentation identified (reference group: spouse only)**				
	Both child and spouse	1.28 (0.8-2.03)	.3	
	Child only	0.81 (0.49-1.34)	.41	
Admission age		1.01 (1-1.03)		.05
Elixhauser score		0.99 (0.92-1.07)		.81
SOFA^b^ score		1.09 (1.04-1.14)		<.001

^a^Exploratory analyses were conducted to investigate the association between documentation related to family member involvement and goals of care conversations.

^b^SOFA: sequential organ failure assessment score.

### Association Between Family Involvement and LLST

More than a quarter of the patients (274/858, 31.9%) had documented LLST (Table 1). Child only involvement was not associated with LLST (OR 1.49; 95% CI 0.89-2.52; *P*=.13) compared to spouse only involvement. Child plus spouse involvement was associated with higher odds of LLST (OR 1.6; 95% CI 1.02-2.52; *P*=.04) compared to spouse only involvement (Table 3).

**Table 3:** Limitations in life-sustaining therapy conversations^a^.

Variables		Odds ratio (95% CI)	*P* value
**Sex (reference group: male)**			
	Female	0.98 (0.7-1.37)	.91
**Marital status (reference group: married)**			
	Not married	1.51 (0.99-2.28)	.05
	Unknown	1.16 (0.44-3.05)	.77
**Ethnicity (reference group: White)**			
	Other	0.85 (0.57-1.28)	.44
	Unknown	0.6 (0.27-1.36)	.22
**Type of family member documentation identified (reference group: spouse only)**			
	Both child and spouse	1.6 (1.02-2.52)	.04
	Child only	1.49 (0.89-2.52)	.13
Admission age		1.04 (1.03-1.06)	<.001
Elixhauser score		0.96 (0.89-1.03)	.24
SOFA^b^ score		1.15 (1.11-1.2)	<.001

^a^Results of exploratory analyses to investigate the association between documentation related to family member involvement and limitations in life-sustaining therapy.

^b^SOFA: sequential organ failure assessment score

### Association Between Family Involvement and Mortality

Over a third of the patients (342/858, 39.9%) died 3 months post hospital admission (Table 1). Compared to spouse only involvement, we found no significant differences in the 3-month mortality by family member type, including child plus spouse involvement (OR 1.38; 95% CI 0.91-2.09; *P*=.13) and child only involvement (OR 1.47; 95% CI 0.9-2.41; *P*=.12) (Table 4).

**Table 4:** Mortality at 3 months following admission^a^.

Variables			Odds ratio (95% CI)	*P* value
**Sex (reference group: male)**				
	Female	0.76 (0.56-1.05)		.09
**Marital status (reference group: married)**				
	Not married	0.71 (0.47-1.05)		.09
	Unknown	1.01 (0.41-2.51)		.98
**Ethnicity (reference group: White)**				
	Other	0.82 (0.56-1.22)		.33
	Unknown	1.28 (0.62-2.65)		.51
**Type of family member documentation identified (reference group: spouse only)**				
	Both child and spouse	1.38 (0.91-2.09)		.13
	Child only	1.47 (0.9-2.41)		.12
Admission age			1.03 (1.02-1.04)	<.001
Elixhauser score			1.01 (0.95-1.09)	.7
SOFA_b_ score			1.2 (1.15-1.25)	<.001

^a^Results of exploratory analyses to investigate the association between documentation related to family involvement and 3-month mortality since hospital admission.

^b^SOFA: sequential organ failure assessment score.

## Discussion

### Principal Results

This study demonstrated that child plus spouse involvement in decision-making within the first 48 hours of an ICU stay was associated with LLST for mechanically ventilated patients when compared to spouse involvement only. To our knowledge, this is the first study to demonstrate an association between spouse plus child involvement and LLST in mechanically ventilated patients in the ICU. Family members may find it easier to make complex decisions in a group with other family members, and this approach may help in reaching a consensus in the context of a poor prognosis. Prior research has shown that family members take on the end-of-life (EOL) decision-maker role together as a unit and collaborate, and even designated surrogate decision makers prefer to structure the interaction around collaborative group decision-making rather than take on the role individually [14].

Unlike the association found between LLST and family involvement, there was no association between family member involvement and documentation of GOCC. One possible explanation is that a GOCC is defined as a palliative and end-of-life care process measure [40,44], meaning that such conversations are part of evidence-based guidelines and will occur regardless of which family member is present [45]. Meanwhile, LLST is the next step after a GOCC occurs and is important to establish when actually making decisions about life-limiting therapies, which may collectively involve the patients, their family members, and clinicians.

### Comparison With Prior Work

Research has demonstrated that the type of family involvement often varies across racial and ethnic groups and there is a growing number of studies exploring the role of race, ethnicity, and culture in caregiving [36,46,47]. Compared to White patients, we observed that non-White patients had a high proportion of child only involvement. Similarly, previous studies have found that African American patients are more likely to receive assistance from adult children rather than spouses [47-49]. Williams and Dilworth-Anderson examined connections of social support for 187 community-dwelling African American elders and demonstrated that the adult child was the most common type of relationship to the care recipient (62%), surpassing spouse (6%), friend (3%), and other kin (29%) [50]. Similarly, Miller and Guo demonstrated that African American caregivers for persons with dementia were found to be younger, less educated, having lower income, and married for fewer years than White caregivers [51]. Though this study included participants from a single site, which may impact generalizability, the findings demonstrate potential racial and ethnic differences regarding the type of family members involved in care within the ICU setting, but further research is warranted.

Given the rising number of individuals facing serious illness, receiving critical care, and living longer, our study adds to the growing body of knowledge that calls for the need to develop approaches that are tailored to the specific subpopulations of family members who are involved in ICU patient care and decision-making.

### Limitations

This study has several limitations. First, we examined data from 2008 to 2012, so our findings may not be generalizable to the more recent years. Second, the cross-sectional nature of the study did not enable us to assess causality or temporality between family involvement and patient outcomes. Third, because our sample was limited to clinical notes from a single tertiary care hospital in northeastern United States and lacked racial diversity, our algorithm may not be generalizable to other hospitals, ICU populations, or geographic areas. Fourth, as noted in other studies [34,44,52], our methods were dependent on the quantity and quality of documentation that exist in the EHR, so it is possible that some family-related documentation or actual interaction with and involvement of families may have been missed. Moreover, our models may not fully account for all possible confounders, and we were unable to capture other factors that may impact the relationship between family involvement and patient outcomes. Fifth, we focused on documentation generated within the first 48 hours by nurses, case managers, social workers, physician assistants, and physicians, but critical care is a broad, interdisciplinary specialty. The role of other clinicians’ documentations in describing outcomes in the ICU setting is not known. Future work should examine documentation of family involvement generated by other clinical disciplines and other ICU settings. Finally, we used rule-based NLP models, which only detect phrases in notes if they match the specified keywords.

### Conclusions

This study fills an important gap in our understanding of family involvement in patient care and decision-making early in ICU stays. Findings suggest that better decisions about LLSTs will be made if additional family members are engaged, and clinicians should seek out everyone who may want to or need to participate.

## Appendix

Multimedia Appendix 1 Keyword library. This appendix presents the exact phrases used in the keyword bank. It includes an extensive list to account for the wide variations in describing spouse and child. For example, spouse is described as husband, wife, fiancé, girlfriend, boyfriend, companion, partner, spouse, comate, etc. Child is described as son, daughter, grandchild, teenage, girl, boy, child, children, grandson, granddaughter, etc.

Multimedia Appendix 2 Keywords used in relevant context. This Multimedia Appendix provides examples of how the keywords found in the clinical notes were used in relevant context in the analysis.

Multimedia Appendix 3 Keywords used in nonrelevant context. This Multimedia Appendix provides examples of how the keywords found in the clinical notes were used in a nonrelevant context and eliminated during the analysis.

## Abbreviations

BIDMC: Beth Israel Deaconess Medical Center
EHR: electronic health record
GOCC: goals of care conversation
ICU: intensive care unit
LLST: limitations in life-sustaining therapy
LOS: length of stay
MICU: medical intensive care unit
MIMIC: Medical Information Mart for Intensive Care
MIT: Massachusetts Institute of Technology
NLP: natural language processing
SNF: skilled nursing facility
SOFA: sequential organ failure assessment score
