# Supplementary material for: Associations Between Family Member Involvement and Outcomes of Patients Admitted to the Intensive Care Unit: Retrospective Cohort Study
Source: JMIR Med Inform. 2022 Jun 15;10(6):e33921. doi: 10.2196/33921 (PMC9244649; doi:10.2196/33921)
Supplement: Multimedia Appendix 1 [file medinform_v10i6e33921_app1.pdf]

Keyword Library

Wife	Wifes	Wife's	Wifes'
Wive	Wives	Wive's	Wives'
Husband	Husbands	Husband's	Husbands'
Partner	Partners	Partner's	Partners'
Spouse	Spouses	Spouse's	Spouses'
Significant other	Significant other's	Significant others'	Girlfriend
Girlfriends	Girlfriend's	Girlfriends'	Boyfriend
Boyfriends	Boyfriend's	Boyfriends'	Fiance's
Fiances	Fiancé	Fiancee's	Fiancees'
Ex	Exes	Exe's	Exes'
Exs	Ex's	Exs'	Ex-husband
Ex-husbands'	Ex-wife	ex-wifes	Ex-wife's
Ex-wifes'	Ex-wive	Ex-wives	Ex-wives'
Ex-wive's	Ex-partner	Ex-husband	Exhusbands
Exhusband's	Exhusbands'	Exwife	Exwifes
Exwife's	Exwifes'	Exwive	Ex-wives
Exwive's	Exwives'	Companion	Companions
Companion's	Companions'	Mate	Helpmate
Confidant	Intimate	Sweetheart	Comate
Betterhalf	Better half	Spousal	Newlywed
Newlyweds	Newlywed's		

Child	Childs	Child's	Childs'
Children	Children	Childrens	Children's
Childrens'	Son	Sons	Son's
Sons'	Daughter	Daughters	Daughter's
Daughters'	Dtr	Kid	Kids
Kid's	Kids'	Step-child	Stepchild
Step-daughter	Stepdaughter	Step-son	Stepson
Adopted son	Adopted daughter	Adopted child	Grandchild
Grandchilds	Grandchild's	Grandchilds'	Grandchildren
Grandchildrens	Grandchildren's	Grandchildrens'	Grandson
Granddaughter	Grandkid	Teen	Teens
Teen's	Teens'	Teenager	Teenagers
Teenager's	Teenagers'	Boy	Boys
Boy's	Boys'	Girl	Girls
Girl's	Girls'	16-y/o	30 year old
30 year-old	30-year old	30-year-old	Offspring
Offsprings	Descendent	Descendents	Young one
Little one	Young ones	Little ones	Youngster
Youth	Youngsters	Juvenile	Grandkids
Grandkids	Young folk	Young folks	Daugh
Daught	Adolescent	Adolescents	Adolescents'
Adolescent's	Year-olds	Year olds	Daughter-in-law
Son-in-law	Daughter in law	Son in law	

Reference:

This is a Multimedia Appendix to a full manuscript published in the J Med Internet Res. For full copyright and citation information see http://dx.doi.org/10.2196/jmir.33921
