# Supplementary material for: Associations Between Family Member Involvement and Outcomes of Patients Admitted to the Intensive Care Unit: Retrospective Cohort Study
Source: JMIR Med Inform. 2022 Jun 15;10(6):e33921. doi: 10.2196/33921 (PMC9244649; doi:10.2196/33921)
Supplement: Multimedia Appendix 2 [file medinform_v10i6e33921_app2.pdf]

Keywords Used in Relevant Context

Domain	**Sample phrases from clinical notes**
Spousal Involvement	Family meeting **met with husband**, cousin sister, RN, and gyn service
	**Wife** has been updated frequently
	**SW met with pt's wife** at bedside this evening
	ICU consent **signed by wife**
	HCP is **wife**
Child Involvement	**DNR confirmed with daughter**
	Full code **discussed with patient's son**
	Health care proxy: **daughter**
	Full code, **confirmed with son**
	DNR, okay to intubate Communication: Patient, **Son**

Reference:

This is a Multimedia Appendix to a full manuscript published in the J Med Internet Res.
For full copyright and citation information see http://dx.doi.org/10.2196/jmir.33921
