# Supplementary material for: Associations Between Family Member Involvement and Outcomes of Patients Admitted to the Intensive Care Unit: Retrospective Cohort Study
Source: JMIR Med Inform. 2022 Jun 15;10(6):e33921. doi: 10.2196/33921 (PMC9244649; doi:10.2196/33921)
Supplement: Multimedia Appendix 3 [file medinform_v10i6e33921_app3.pdf]

Keywords Used in Non-Relevant Context

Domain	**Sample phrases from clinical notes**
Spousal Involvement	Lives with wife
	His wife has a hx of hep B
	Patient is married and lives with husband
	his male partner smokes
	Husband passed away a few years ago
Child Involvement	RR mid teens even and unlabored
	Married, no children
	Pt had a 6 year old son
	Son had renal CA
	2 daughters, son lives downstairs

Reference:

This is a Multimedia Appendix to a full manuscript published in the J Med Internet Res.
For full copyright and citation information see http://dx.doi.org/10.2196/jmir.33921
